# *Enterobacter oligotrophica* sp. nov., a novel oligotroph isolated from leaf soil

**DOI:** 10.1002/mbo3.843

**Published:** 2019-05-07

**Authors:** Hironaga Akita, Akinori Matsushika, Zen‐ichiro Kimura

**Affiliations:** ^1^ Research Institute for Sustainable Chemistry National Institute of Advanced Industrial Science and Technology (AIST) Higashi‐Hiroshima Japan; ^2^ Graduate School of Advanced Sciences of Matter Hiroshima University Higashi‐Hiroshima Japan; ^3^ Department of Civil and Environmental Engineering National Institute of Technology Kure College Kure Japan

**Keywords:** average nucleotide identity value analysis, *Enterobacter*, genome sequence, multilocus sequence analysis, oligotroph, Voges–Proskauer test

## Abstract

A novel oligotrophic bacterium, designated strain CCA6, was isolated from leaf soil collected in Japan. Cells of the strain were found to be a Gram‐negative, non‐sporulating, motile, rod‐shaped bacterium. Strain CCA6 grew at 10–45°C (optimum 20°C) and pH 4.5–10.0 (optimum pH 5.0). The strain was capable of growth in poor‐nutrient (oligotrophic) medium, and growth was unaffected by high‐nutrient medium. The major fatty acid and predominant quinone system were C_16:0_ and ubiquinone‐8. Phylogenetic analysis based on 16S rRNA gene sequences indicated strain CCA6 presented as a member of the family *Enterobacteriaceae*. Multilocus sequence analysis (MLSA) based on fragments of the *atpD*,* gyrB*,* infB*, and *rpoB* gene sequences was performed to further identify strain CCA6. The MLSA showed clear branching of strain CCA6 with respect to *Enterobacter* type strains. The complete genome of strain CCA6 consisted of 4,476,585 bp with a G+C content of 54.3% and comprising 4,372 predicted coding sequences. The genome average nucleotide identity values between strain CCA6 and the closest related *Enterobacter* type strain were <88.02%. Based on its phenotypic, chemotaxonomic and phylogenetic features, strain CCA6 (=HUT 8142^T^ =KCTC 62525^T^) can be considered as a novel species within the genus *Enterobacter* with the proposed name *Enterobacter oligotrophica*.

## Graphical abstract

In this study, we report a novel strain isolated from the leaf soil collected in Japan. Interesting features of this isolate were its growth unaffected by high‐nutrient medium and growth potential in poor‐nutrient (oligotrophic) medium. We performed physiological, chemotaxonomic, and phylogenetic analyses. Based on the results we propose the isolate represents a novel species within the genus *Enterobacter*.

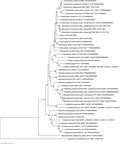

## 1 INTRODUCTION

A variety of microorganisms are used in industrial fermentation for production of enzymes, medicines, and other organic compounds. Those microorganisms are generally grown in high‐nutrient medium containing large amounts of sugar, nitrogen, phosphorus, minerals, and other nutrients that are considered essential for their growth. Consequently, nutrient cost is an important factor when trying to achieve cost‐effective fermentation. This has led to the development of several related technologies, including bioengineering of microorganisms so as to enhance their productivity and yield (Min, Hwang, Lim, & Jung, 2017), use of agricultural byproducts as carbon and mineral sources (Thomsen, 2005), and production of chemicals such as bioemulsifiers (Banat, Satpute, Cameotra, Patil, & Nyayanit, 2014), biofuels (Ho, Ngo, & Guo, 2014), and biosurfactants (Banat et al., 2014) from renewable substrates.

Oligotrophs are organisms that grow under conditions of low levels of nutrients but grow more slowly at high levels (Kuznetsov, Dubinina, & Lapteva, 1979). Consequently, oligotrophs have not been applied for industrial use. We suggest that production costs could be reduced if oligotrophs could be used for industrial fermentation, and therefore screened for oligotrophs that are unaffected by a high‐nutrient condition. Here, we report the screening, isolation, and characterization of an oligotrophic bacterium from leaf soil, which is one kind of the compost and is accrued by fermenting the dry leaves. The isolate was named strain CCA6. This bacterium was capable of growth on poor‐nutrient medium, and its growth was unaffected by high‐nutrient mixtures. Moreover, physiological, chemotaxonomic, and phylogenetic analyses as well as average nucleotide identity (ANI) value analysis were performed to characterize strain CCA6. Based on the results of these analyses, we propose that strain CCA6 represents a novel species within the genus *Enterobacter*, for which the name *E. oligotrophica* sp. nov. is proposed.

## 2 MATERIALS AND METHODS

### 2.1 Bacterial isolation

Soil samples were collected from Higashi‐Hiroshima city in Hiroshima prefecture, Japan. A 1.5% agar (Nacalai tesque, Kyoto, Japan) plate (pH 7.2), which contained sulfates (>0.4%), calcium (>0.1%), iron (>0.01%), and a few fatty acids and/or other minerals at concentrations <0.01% was used for isolation. After 1 ml of a 10% (w/v) soil wash solution was inoculated onto a plate, the plate was incubated for 2 days at 37°C. Thereafter, a single colony was successively re‐streaked onto a new 1.5% agar plate at least three times to obtain a pure colony. The purified strain was then grown aerobically at 37°C in Nutrient Broth (Kyokuto, Tokyo, Japan) and preserved at −20°C as a suspension in Nutrient Broth supplemented with glycerol (30%, w/v).

### 2.2 Physiological characterization

Growth of strain CCA6 in Nutrient Broth was evaluated at various temperatures (4–50°C), pH (4.0–10.5), and NaCl concentrations (1–7%, w/v), and in the presence of selected antibiotics (ampicillin, chloramphenicol, and kanamycin). The OD_600_, which reflects cell growth, was measured by monitoring the difference between cellular and cell‐free turbidity values using an Eppendorf BioSpectrometer (Eppendorf, Hamburg, Germany). Carbon source utilization was assessed using API 20E (bioMérieux, Marcy‐l'Etoile, France) and API 50 CHE (bioMérieux) according to the manufacturer's instructions. Voges–Proskauer (VP) test was carried out using RapiD 20E (bioMérieux). Enzyme activities were evaluated using API ZYM (bioMérieux).

### 2.3 Chemotaxonomic analyses

The cellular fatty acid composition of strain CCA6 was determined using Sherlock Microbial Identification System Version 6.0 (MIDI, Newark, DE) with TSBA6 database (MIDI). Using the method of Bligh and Dyer (1959), lipids were extracted from lyophilized cells of strain CCA6 and loaded onto a Sep‐Pak Plus Silica cartridge (Waters, Milford, MA). The cartridge was then washed and the quinones were eluted. The quinones were quantified using an ACQUITY UPLC system (Waters) with an Eclipse Plus C18 column (Agilent technologies, Santa Clara, CA). The chromatographic conditions were as follows: mobile phase, methanol/isopropanol (3:1 v/v); flow rate, 0.5 ml/min; column oven temperature, 35°C. The quinone forms were identified as previously described (Tamaoka, Katayama‐Fujimura, & Kuraishi, 1983).

### 2.4 Phylogenetic analysis based on 16S rRNA gene

After strain CCA6 was cultured aerobically for 6 hr at 37°C in Nutrient Broth, the cells were harvested by centrifugation, and their genomic DNA was extracted and purified using an illustra bacteria genomicPrep Mini Spin Kit (GE Healthcare, Chicago, IL) according to the manufacturer's instructions. The 16S rRNA gene was amplified using KOD plus DNA Polymerase (TOYOBO, Osaka, Japan) with the bacterial universal primers 27f (5′‐AGAGTTTGATCMTGGCTCAG‐3′; Lane, 1991) and 1391r (5′‐GACGGGCGGTGTGTRCA‐3′; Turner, Pryer, Miao, & Palmer, 1999). After purifying the amplified PCR product using a Wizard SV Gel and PCR Clean‐up System (Promega, Madison, WI), the purified product was cloned into pTA2 vector (TOYOBO) and sequenced. Sequence was then compared with reference sequences available in the GenBank/EMBL/DDBJ databases using BLAST. Multiple alignment and construction of a maximum‐likelihood tree were performed using MEGA‐X (Kumar, Stecher, Li, Knyaz, & Tamura, 2018) with Tamura and Nei model (1993).

### 2.5 Multilocus sequence analysis based on housekeeping genes

Multilocus sequence analysis (MLSA) was performed using the method of Brady et al. (2008), Brady, Cleenwerck, Venter, Coutinho, and De Vos (2013) with some modifications. A phylogenetic tree of concatenated sequences (2,637 bp), including partial sequences of four housekeeping genes [*atpD* (β subunit of ATP synthase; 642 bp), *gyrB* (DNA gyrase; 743 bp), *infB* (translation initiation factor 2; 615 bp), and *rpoB* (β subunit of RNA polymerase; 637 bp)] from strain CCA6, was also reconstructed using the maximum‐likelihood method with Tamura and Nei model (1993). The housekeeping genes of strain CCA6 and the related type strains are available in the GenBank/DDBL/EMBL databases.

### 2.6 Genome sequencing and ANI value analysis

The concentration and purity of the genomic DNA were measured using a NanoDrop ND‐1000 spectrophotometer (Thermo Fisher Scientific, Waltham, MA) and a Quant‐iT dsDNA BR assay kit (Invitrogen, Waltham, MA), respectively. After fragmenting the genomic DNA (20.9 μg) into approximately 20‐kb pieces using g‐TUBE (Covaris, Brighton, UK), the resultant fragments were ligated to SMRTbell sequencing adapters using a SMRTbell Template Prep Kit 1.0 (Pacific Biosciences, Menlo Park, CA), yielding SMRTbell libraries. The library size was measured using Agilent 2200 TapeStation (Agilent Technologies, Santa Clara, CA). The SMRTbell libraries were then bound to polymerases and sequencing primers using a DNA/Polymerase binding kit P6 v2 (Pacific Biosciences), yielding the sequencing templates. The concentration of the sequencing templates was calculated using Binding Calculator v2.3.1.1 (Pacific Biosciences), after which the templates were bound to MagBeads using a MagBead kit (Pacific Biosciences) and loaded onto SMRT Cells 8Pac v3 (Pacific Biosciences). Sequencing was then performed using PacBio RS II (Pacific Biosciences).

The raw data included 100,771 reads with 330 coverage and were assembled de novo using SMRT Analysis v2.3.0 (Pacific Biosciences; Chin et al., 2013) to filter the subreads. Genome annotation was performed using CRITICA (Badger & Olsen, 1999) and Glimmer2 (Delcher, Harmon, Kasif, White, & Salzberg, 1999). The tRNA and rRNA genes were detected using tRNAScan‐SE (Lowe & Eddy, 1997) and BLASTN (Altschul, Gish, Miller, Myers, & Lipman, 1990), respectively. ANI values were calculated through pairwise genome comparison of whole‐genome sequences of strain CCA6 and its related *Enterobacter* type strains using the ANI algorithm (Goris et al., 2007) implemented within OrthoANIu tools (Yoon, Ha, Lim, Kwon, & Chun, 2017).

The genome properties of type strains of *Enterobacter*,* Klebsiella*,* Kosakonia*,* Lelliottia*,* Pluralibacter*,* Pseudescherichia*,* Pseudomonas*, and *Raoultella* species are presented in Table A1.

## 3 RESULTS AND DISCUSSION

### 3.1 Isolation of strain CCA6

**Figure A1:**
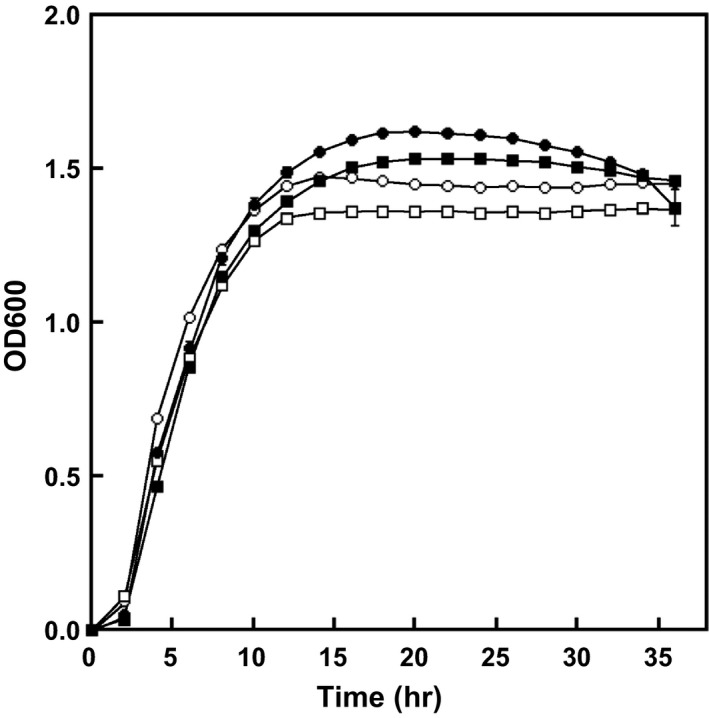
Growth rates at 30°C of strain CCA6 and *E. coli* MG1655. The results for strain CCA6 and *E*. *coli* MG1655 are shown as filled and open symbols, respectively. The media are indicated as follows: circles, Nutrient Broth (pH 7.0) and squares, LB media (pH 7.0). The OD_600_ was measured using a Bio Microplate Reader HiTS (Scinics, Tokyo, Japan). Experiments were performed in triplicate

To obtain oligtrophic microorganisms, filtrates were prepared from several soil samples and plated onto 1.5% agar (pH 7.2) without a carbon source or other medium components. After incubation for 2 days at 37°C, a single colony was obtained from the leaf soil filtrate. A purified colony was then obtained through standard dilution plating on the same plates and was named strain CCA6. Although high‐nutrient mixtures suppress the growth of some oligotrophic bacteria (Ohta, 2000; Ohta & Taniguchi, 1988), strain CCA6 showed a higher rate of growth, similar to that of *Escherichia coli* MG1655, when cultured in Nutrient Broth or LB media (Figure A1). By contrast, *E. coli* MG1655 did not grow on a 1.5% agar (pH 7.2). These results suggest we had successfully isolated the desired oligotroph.

### 3.2 Morphological and physiological characterization

**Figure A2:**
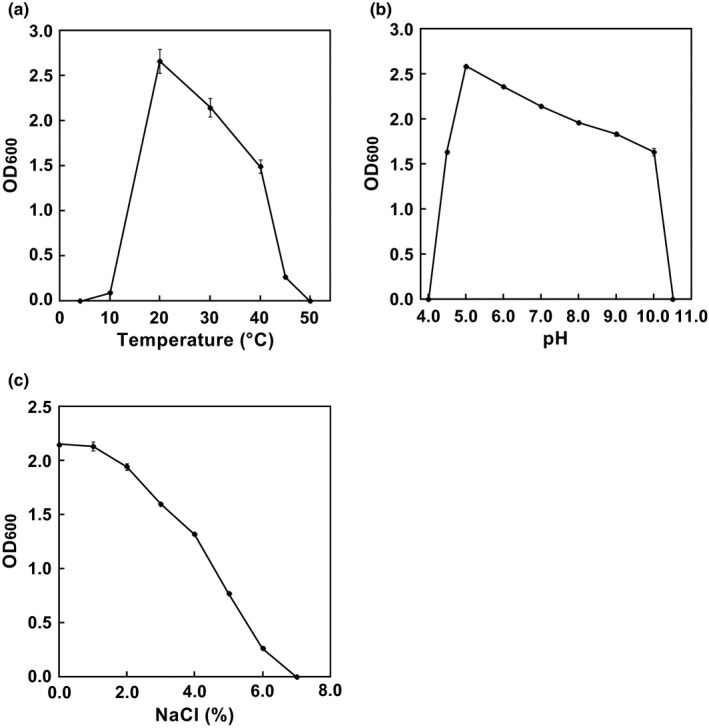
Effects of culture conditions on growth of strain CCA6. (a) Effects of culture temperature. Cells were cultured in Nutrient Broth (pH 7.0). (b) Effects of culture pH. Cells were cultured in Nutrient Broth at 30°C. (c) Effect of NaCl concentration. Cells were cultured in Nutrient Broth (pH 7.0) at 30°C. Error bars indicate SE (*n* = 3)

Cells of strain CCA6 were Gram‐negative, motile, rod‐shaped and non‐sporulating. Colonies grown on Nutrient Broth plates were circular, smooth, glistening, light yellow, and 5.0 mm in diameter after incubation overnight at 37°C. When we examined the effect of culture temperature and pH, we found that the strain was capable of growing at temperatures between 10 and 45°C, but no growth was seen at 4 or 50°C (Figure A2a). The strain also grew effectively at pHs between 4.5 and 10.0, but growth rates were sharply lower at pHs below 4.0 or above 10.5 (Figure A2b). The strain was tolerant to 6% (w/v) NaCl (Figure A2c) and was resistant to ampicillin, but chloramphenicol and kanamycin inhibited its growth.

Strain CCA6 showed a broad range of enzyme activities, including acid phosphatase, *N*‐acetyl‐β‐d‐glucosaminidase, alkaline phosphatase, cystine aminopeptidase, esterase lipase (C8), α‐galactosidase, β‐galactosidase, α‐glucosidase, β‐glucosidase, leucine aminopeptidase, α‐mannosidase, naphthol AS‐BI phosphate, trypsin, and valine aminopeptidase. By contrast, strain CCA6 did not exhibit α‐chymotrypsin, esterase (C4), α‐fucosidase, β‐glucuronidase, or lipase (C14) activity. These results suggest that strain CCA6 is capable of catabolizing a variety of different carbon sources. Culture with different carbon sources revealed that CCA6 was able to utilize the following compounds as a carbon source for growth: inulin, amygdalin, arbutin, esculin ferric citrate, 2‐nitrophenyl β‐d‐galactopyranoside, d‐cellobiose, d‐trehalose, d‐maltose, d‐lactose, d‐galactose, d‐glucose, l‐sorbose, d‐fructose, d‐mannose, l‐rhamnose, dulcitol, d‐sorbitol, d‐mannitol, *N*‐acetyl‐glucosamine, methyl‐β‐d‐xylopyranoside, d‐arabinose, l‐arabinose, d‐xylose, l‐xylose, l‐fucose, d‐lyxose, d‐ribose, adonitol, erythritol, glycerol, l‐arginine, l‐lysine, l‐ornithine, l‐tryptophane, 2‐keto gluconate, citrate, gluconate, pyruvate, and urea. By contrast, no growth occurred on glycogen, gelatin, starch, salicin, d‐melezitose, d‐raffinose, gentiobiose, d‐sucrose, d‐melibiose, d‐turanose, d‐tagatose, inositol, methyl‐α‐d‐glucopyranoside, methyl‐α‐d‐mannopyranoside, d‐fucose, d‐arabitol, l‐arabitol, xylitol, 5‐keto gluconate, or thiosulfate. Differences in phenotypic characteristics of strain CCA6 and its related type species are shown in Table 1.

**Table 1:** Differential characteristics of strain CCA6 and phylogenetically related species

Characteristic	1	2	3	4	5	6	7	8	9	10
Carbon source utilization										
d‐Sucrose	‐	++	++	++	++	++	++	++	++	++
d‐Melibiose	‐	+	++	++	W	++	++	++	++	++
d‐Turanose	‐	+	+	‐	W	+	‐	+	W	+
l‐Rhamnose	++	+	++	‐	++	++	++	++	++	++
Inositol	‐	+	++	++	‐	W	W	+	++	++
Dulcitol	+	+	W	‐	++	‐	‐	+	+	+
d‐Sorbitol	++	++	++	++	W	++	++	++	++	++
Methyl‐α‐d‐glucopyranoside	‐	++	++	++	++	+	+	++	++	++
d‐Arabinose	++	+	++	++	++	++	++	+	++	+
l‐Fucose	++	+	+	‐	++	+	++	+	‐	W
d‐Lyxose	++	++	W	++	+	++	++	++	+	++
Adonitol	+	+	+	‐	W	W	++	+	‐	+
d‐Arabitol	‐	+	+	‐	W	W	++	+	‐	+
2‐Keto gluconate	+	+	++	+	W	‐	+	+	W	++
Enzyme activity										
Arginine dihydrolase	+	++	++	++	++	++	++	‐	++	++
Ornithine decarboxylase	+	++	++	++	++	++	++	‐	++	++
Lysine decarboxylase	+	‐	‐	‐	‐	‐	‐	‐	‐	W
Esculin hydrolysis	+	++	+	++	‐	W	W	+	W	+
Voges–Proskauer test	‐	++	++	++	++	++	++	++	++	++

Note

Strains: 1, strain CCA6; 2, *E. asburiae* ATCC 35953^T^; 3, *E*. *cloacae* subsp. *cloacae* ATCC13047^T^; 4, *E*. *cloacae* subsp. *dissolvens* ATCC 23373^T^; 5, *E*. *hormaechei* subsp. *hormaechei* ATCC 49162^T^; 6, *E*. *hormaechei* subsp. *oharae* DSM 16687^T^; 7, *E*. *hormaechei* subsp. *steigerwaltii* DSM 16691^T^; 8, *E*. *hormaechei* subsp. *xiangfangensis* LMG 27195^T^; 9, *E*. *kobei* ATCC BAA‐260^T^; 10, *E*. *ludwigii* EN‐119^T^. ++, strong positive; +, positive; W, weak positive; —, not detected.

Nearly all *Enterobacter* species produce acetoin as the end product of glucose metabolism, which yields a red complex in the VP test medium. The related *Enterobacter* type strains show a positive VP test; however, strain CCA6 was negative (Table 1).

### 3.3 Chemotaxonomic characterization

When strain CCA6 was cultured aerobically in Nutrient Broth, the major fatty acids were C_16:0_ and summed feature 8 (comprising C_18:1_ω6*c* and/or C_18:1_ω7*c*). The overall fatty acid profile of strain CCA6 was similar to that of *E. hormaechei* subsp. *hormaechei* ATCC 49162^T^ (Table 2). Respiratory quinone analysis showed the presence of ubiquinone‐7 (4.2%), ubiquinone‐8 (87.2%), and menaquinone‐8 (8.6%).

**Table 2:** Comparative fatty acid contents (%) of strain CCA6 and phylogenetically related reference strains

Fatty acids	1	2	3	4	5	6
Saturated fatty acids						
C_10 : 0_	0.04	‐	‐	‐	‐	‐
C_11 : 0_	0.11	‐	‐	0.08	‐	‐
C_12 : 0_	3.7	3.03	2.47	2.50	2.26	3.96
C_13 : 0_	1.4	0.72	0.61	1.18	0.37	0.90
C_14 : 0_	5.6	6.46	6.74	8.39	9.73	6.18
C_16 : 0_	22.7	27.89	29.27	21.75	30.16	25.71
C_17 : 0_	4.0	3.73	3.43	4.94	2.02	3.18
C_18 : 0_	0.3	0.47	0.53	0.38	0.47	0.39
Branched‐chain fatty acids						
iso‐C_15 : 0_ 3‐OH	‐	‐	‐	‐	‐	‐
anteiso‐C_19 : 0_	‐	‐	‐	‐	‐	‐
iso‐C_19 : 0_	0.2	‐	0.11	‐	‐	‐
Unsaturated fatty acids						
C_15 : 1_ω6*c*	0.1	‐	‐	‐	‐	‐
C_15 : 1_ω8*c*	0.3	‐	0.09	0.14	‐	‐
C_16 : 1_ω5*c*	‐	0.21	0.21	0.22	‐	0.21
C_17 : 1_ω8*c*	0.4	‐	‐	0.44	‐	‐
11‐methyl‐C_18 : 1_ω7*c*	‐	0.26	‐	‐	‐	‐
C_18 : 1_ω5*c*	‐	‐	0.18	0.19	‐	‐
Hydroxy fatty acids						
C_15 : 0_ 3‐OH	‐	0.16	0.15	0.36	‐	0.21
Cyclopropane acids						
cyclo‐C_17 : 0_	14	26.01	20.4	21.08	21.69	25.17
cyclo‐C_19 : 0_ω8*c*	3.3	7.04	6.10	4.35	5.79	5.99
Summed feature						
1	1.3	0.93	0.64	1.46	0.34	0.74
2	8.1	6.14	6.85	6.21	6.79	6.79
3	12.6	3.51	6.50	5.71	4.49	4.9
8	21.3	13.44	14.27	20.62	15.88	15.68

Note

Strains: 1, strain CCA6; 2, *E. asburiae* ATCC 35953^T^; 3, *E*. *cloacae* subsp. *cloacae* ATCC 13047^T^; 4, *E*. *hormaechei* subsp. *hormaechei* ATCC 49162^T^; 5, *E*. *hormaechei* subsp. *xiangfangensis* LMG 27195^T^; 6, *E*. *ludwigii* EN‐119^T^. Data from 2 to 6 are from Gu, Li, Yang and Huo (2014). —, not detected/not reported. Summed feature 1 consists of iso‐C_15 : 1_ H and/or C_13 : 0_ 3‐OH; Summed feature 2 consists of iso‐C_16:1_ I and/or C_14:0_ 3‐OH and/or C_12:0_ unidentified aldehyde or an unidentified fatty acid with an equivalent chain length of 10.928; Summed feature 3 consists of C_16:1_ω6*c* and/or C_16:1_ω7*c*; summed feature 8 consists of C_18:1_ω6*c* and/or C_18:1_ω7*c*.

### 3.4 Phylogenetic affiliation of strain CCA6

The genus *Enterobacter* was first proposed by Hormaeche and Edwards (1960), and was classified as Gram‐negative, rod‐shaped, motile bacteria. To date, more than 18 *Enterobacter* species have been reported, and the *Enterobacter cloacae* complex has been rearranged in *E. cloacae*,* E. asburiae*,* E. hormaechei*,* E. kobei*,* E. ludwigiii*, and their subspecies based on whole‐genome DNA–DNA hybridizations and phenotypic characteristics (Mezzatesta, Gona, & Stefani, 2012).

**Figure 1:**
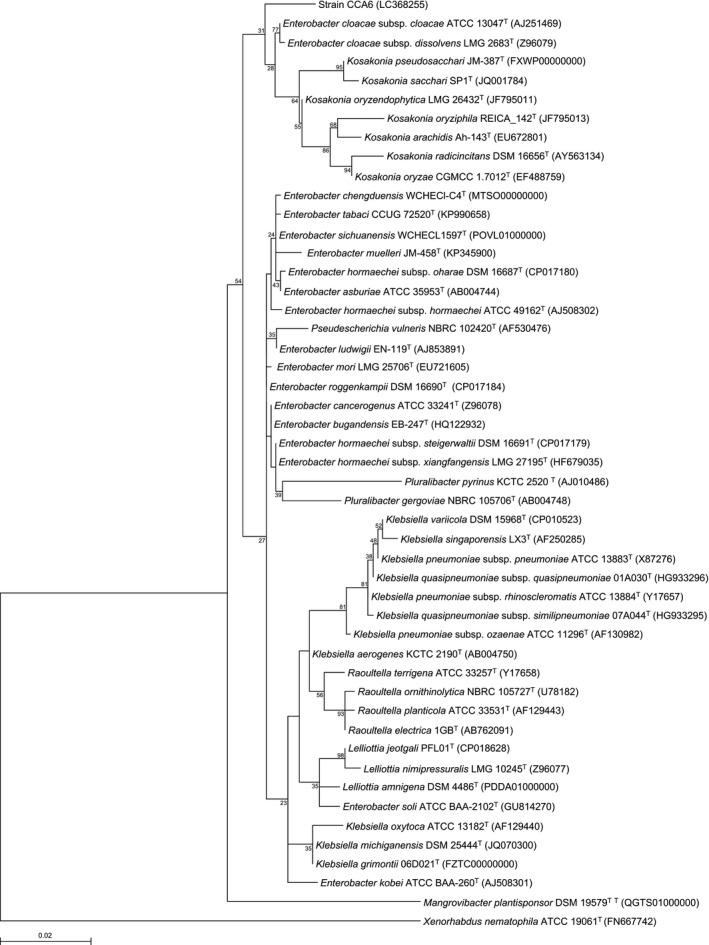
Phylogenetic tree constructed from analysis of 16S rRNA gene sequences showing the relationships between strain CCA6 and the related type strains. The bar indicates a 0.02% nucleotide substitution rate. The tree was rooted using *Xenorhabdus nematophila* ATCC 19061^T^ as the outgroup

To confirm the phylogenetic position of strain CCA6, the 16S rRNA gene sequence (1,294 bp) was determined. In the maximum‐likelihood tree based on almost complete sequences of the 16S rRNA gene, strain CCA6 fell inside the cluster comprising members of the genus *Enterobacter* and *Kosakonia* (Figure 1). The sequences of the following *E. cloacae* complex species showed similarity to that of strain CCA6: *E. cloacae* subsp. *dissolvens* LMG 2683^T^ (98.3%), *E*. *cloacae* subsp. *cloacae* ATCC 13047^T^ (98.0%), *E. sichuanensis* WCHECL1597^T^ (97.8%), *E. chengduensis* WCHECl‐C4^T^ (97.7%), *K. oryzendophytica* LMG 26432^T^ (97.7%), *E. ludwigii* EN‐119^T^ (97.6%), *E. roggenkampii* DSM 16690^T^ (97.6%), and *E. mori* LMG 25706^T^ (97.4%).

**Figure 2:**
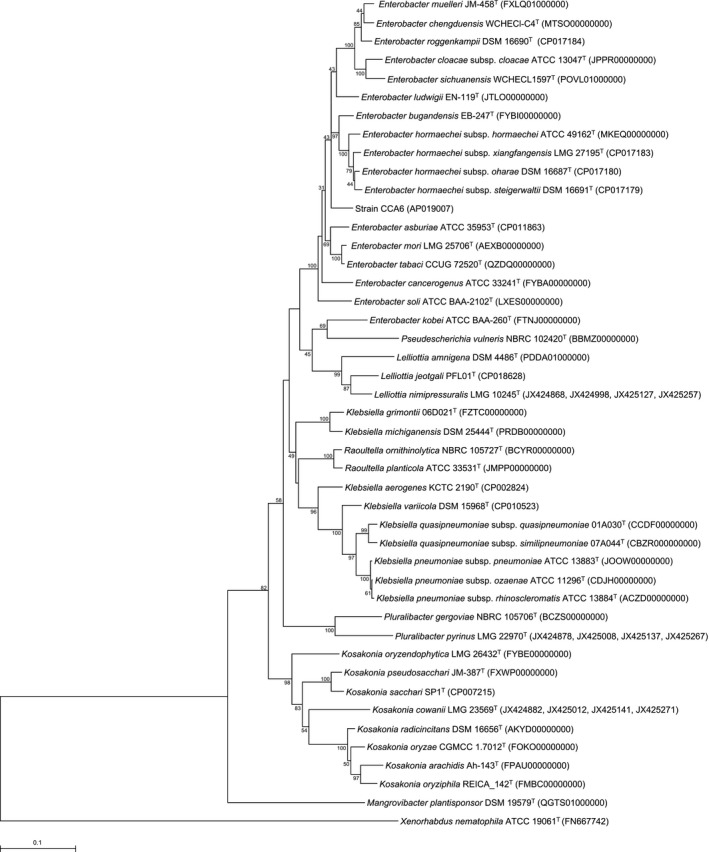
Phylogenetic tree reconstructed from analysis of the sequences of four housekeeping genes (*atpD*,* gyrB*,* infB*, and *rpoB*) and showing the relationships between strain CCA6 and the related type strains. The bar indicates a 0.1% nucleotide substitution rate. The tree was rooted using *X. nematophila* ATCC 19061^T^ as the outgroup

According to Brady et al. (2008, 2013), MLSA is also useful for identification of bacterial species. The MLSA showed that strain CCA6 exhibited similarities of 96.0%, 96.0%, 96.0%, 95.9%, 95.9%, 95.8%, 95.8%, 95.7%, and 95.6% to its closest relatives, *E. bugandensis* EB‐247^T^, *E. hormaechei* subsp. *xiangfangensis* LMG 27195^T^, *E. ludwigii* EN‐119^T^, *E. hormaechei* subsp. *hormaechei* ATCC 49162^T^, *E. hormaechei* subsp. *steigerwaltii* DSM 16691^T^, *E. asburiae* ATCC 35953^T^, *E. hormaechei* subsp. *oharae* DSM 16687^T^, *E. tabaci* CCUG 72520^T^, and *E. mori* LMG 25706^T^, respectively. Moreover, strain CCA6 clusters on its own branch separately from other *Enterobacter* species. (Figure 2).

### 3.5 Genome properties and ANI values

The genome sequence of strain CCA6 was 4,476,585 bp. The G+C content was 54.3%, which fell within range of those of *Enterobacter* type strains (Table S1). Within the genomic DNA of strain CCA6, 4,372 predicted coding sequences were identified. In addition, 85 tRNA genes and 25 rRNA genes were detected.

To carry out a phylogenetic comparison of strain CCA6 and the related species in the family *Enterobacteriaceae*, ANI values were calculated (Table S1). The ANI values between strain CCA6 and the related type strains belonging to the genera *Klebsiella*,* Kosakonia*,* Pluralibacter*,* Pseudescherichia*, and *Raoultella* were all <79.70%. Moreover, the ANI values between strain CCA6 and the related *Enterobacter* type strains were in the range of 79.75–88.02%, which was clearly below the cutoff of 95–96% for prokaryotic species delineation as established by Richter and Rosselló‐Móra (2009).

## 4 CONCLUSION

We have isolated a Gram‐negative, non‐sporulating, rod‐shaped bacterium from leaf soil collected in Japan, which was designated strain CCA6. 16S rRNA gene sequence analysis revealed that strain CCA6 presented as a member of the family *Enterobacteriaceae*. (Figure 1). Moreover, MLSA based on partial sequences of the *atpD*,* gyrB*,* infB*, and *rpoB* gene showed clear separation between strain CCA6 and the related *Enterobacter* type strains (Figure 2). The ANI values between strain CCA6 and its closely related type strains were <88.02% (Table S1). Interesting features of strain CCA6 were its growth potential in oligotrophic medium and the fact that its growth was unaffected by high‐nutrient media. Strain CCA6 therefore has potential for utilization as a host bacterium for industrial fermentation of valuable compounds. Although the related *Enterobacter* type strains are capable of utilizing disaccharides such as d‐sucrose and d‐turanose, strain CCA6 did not catabolize those disaccharides (Table 1). When cellular fatty acids were compared between strain CCA6 and the related *Enterobacter* type strains, we found that fatty acids C_16:0_ and summed feature 8 occur in most members of the related *Enterobacter* type strains. By contrast, the ratio of C_11:0_, C_15:1_ω8*c*, C_17:1_ω8*c*, and iso‐C_19:0_ in strain CCA6 was significantly higher than in the close relatives, and the fatty acid C_15:1_ω6*c* was only detected in strain CCA6 (Table 2).

Based on its phylogenetic, phenotypic, and chemotaxonomic features, strain CCA6 can be considered as a novel species in the genus *Enterobacter*, which we propose to name *E. oligotrophica* sp. nov.

### 4.1 Description of *E. oligotrophica* sp. nov

*Enterobacter oligotrophica* (o.li.go.tro'phi.ca. Gr. adj. oligos little; Gr. adj. trophikos nursing, tending or feeding; N.L. fem. adj. *oligotrophica* eating little, referring to a bacterium living on low‐nutrient media).

Cells are aerobic, Gram‐negative, non‐sporulating, and rod‐shaped (1.0–2.0 μm × 4.0–5.0 μm). Colonies are circular, smooth, glistening, light yellow, and grow to 5.0 mm in diameter on Nutrient Broth plates after incubation for 24 hr at 37°C. Growth is observed in poor‐nutrient medium, and growth is unaffected by high‐nutrient medium. The VP test is negative. The major cellular fatty acids are C_16:0_ and sums of C_16 : 1_ω6*c* and/or C_16 : 1_ω7*c* or C_18 : 1_ω6*c* and/or C_18 : 1_ω7*c*. The predominant quinone system is ubiquinone‐8. Growth is observed in Nutrient Broth at 10–45°C and pH 4.5–10.0, with optimal growth at 20°C and pH 5.0. Growth occurs in the presence of 0–6% (w/v) NaCl as well as ampicillin. Strain CCA6 is positive for lysine decarboxylase. No growth occurs on d‐sucrose, d‐melibiose, d‐turanose, d‐tagatose, inositol, or methyl‐α‐d‐glucopyranoside. Strain CCA6 is clearly separated from the related *Enterobacter* type strains by MLSA based on partial sequences of the *atpD*,* gyrB*,* infB*, and *rpoB* gene. The genome size of the type strain is 4,476,585 bp, which has a G+C content of 54.3%.

## CONFLICT OF INTERESTS

None declared.

## AUTHOR CONTRIBUTIONS

HA an ZK designed, carried out the experiments, and wrote the manuscript. AM revised the manuscript.

## ETHICS STATEMENT

None required.

## Data Availability

The 16S rRNA gene sequence of strain CCA6 is available in the GenBank/EMBL/DDBJ databases under accession number LC368255. The complete genome sequence of strain CCA6 has been deposited in the DDBJ/EMBL/GenBank under accession number AP019007. The type strain is CCA6^T^ and was deposited in two international strain collection institutes with the following accession numbers: HUT 8142^T^ = KCTC 62525^T^.

## APPENDIX 1

### 1.1

**Table A1:** Genome properties of type strains of *Enterobacter*,* Klebsiella*,* Kosakonia*,* Lelliottia*,* Pluralibacter*,* Pseudescherichia*,* Pseudomonas*, and *Raoultella* species used in this study

Strains	Size (bp)	G+C (mol%)	Protein	Accession no.
*Enterobacter*				
* E*. *asburiae* ATCC 35953^T^	4,713,742	55.4	4,436	CP011863
* E*. *bugandensis* EB‐247^T^	4,971,744	56.0	4,344	FYBI00000000
* E*. *cancerogenus* ATCC 33241^T^	4,879,939	55.6	4,521	FYBA00000000
* E*. *chengduensis* WCHECl‐C4^T^	5,138,130	55.7	4,745	MTSO00000000
* E*. *cloacae* subsp. *cloacae* ATCC 13047^T^	5,551,574	54.6	5,393	JPPR00000000
* E*. *hormaechei* subsp. *hormaechei* ATCC 49162^T^	4,890,213	55.2	4,522	MKEQ00000000
* E*. *hormaechei* subsp. *oharae* DSM 16687^T^	4,724,316	55.6	4,390	CP017180
* E*. *hormaechei* subsp. *steigerwaltii* DSM 16691^T^	4,782,480	55.6	4,424	CP017179
* E*. *hormaechei* subsp. *xiangfangensis* LMG 27195^T^	4,661,849	55.3	4,306	CP017183
* E*. *kobei* ATCC BAA‐260^T^	4,700,329	55.5	4,424	FTNJ00000000
* E*. *ludwigii* EN‐119^T^	4,952,770	54.6	4,459	JTLO00000000
* E*. *mori* LMG 25706^T^	4,953,765	55.3	4,496	AEXB00000000
* E*. *muelleri* JM‐458^T^	4,695,678	55.9	4,423	FXLQ01000000
* E*. *roggenkampii* DSM 16690^T^	4,748,414	56.0	4,451	CP017184
* E*. *sichuanensis* WCHECL1597^T^	4,897,201	55.2	4,634	POVL01000000
* E*. *soli* ATCC BAA‐2102^T^	4,960,767	53.8	4,571	LXES00000000
* E*. *tabaci* CCUG 72520^T^	4,927,887	55.5	4,627	QZDQ00000000
*Klebsiella*				
* K. aerogenes* KCTC 2190^T^	5,280,350	54.8	4,912	CP002824
* K. grimontii* 06D021^T^	6,168,876	55.4	5,986	FZTC00000000
* K. michiganensis* DSM 25444^T^	6,193,009	56.0	5,732	PRDB00000000
* K. pneumoniae* subsp. *ozaenae* ATCC 11296^T^	4,925,250	57.2	4,458	CDJH00000000
* K. pneumoniae* subsp. *pneumoniae* ATCC 13883^T^	5,544,684	57.0	5,205	JOOW00000000
* K. pneumoniae* subsp. *rhinoscleromatis* ATCC 13884^T^	5,280,675	56.9	5,671	ACZD00000000
* K. quasipneumoniae* subsp. *quasipneumoniae* 01A030^T^	5,465,736	58.0	5,287	CCDF00000000
* K. quasipneumoniae* subsp. *similipneumoniae* 07A044^T^	5,109,717	58.2	4,927	CBZR000000000
* K. variicola* DSM 15968^T^	5,521,203	57.6	5,200	CP010523
*Kosakonia*				
* K. arachidis* Ah‐143^T^	5,135,597	52.5	4,861	FPAU00000000
* K. oryzae* CGMCC 1.7012^T^	5,380,462	54.0	4,980	FOKO00000000
* K. oryzendophytica* LMG 26432^T^	4,878,776	53.7	4,459	FYBE00000000
* K. oryziphila* REICA_142^T^	4,814,900	52.7	4,667	FMBC00000000
* K. pseudosacchari* JM‐387^T^	4,956,546	53.9	4,638	FXWP00000000
* K. radicincitans* DSM 16656^T^	5,817,639	53.7	5,660	AKYD00000000
* K. sacchari* SP1^T^	4,902,027	53.7	4,545	CP007215
*Lelliottia*				
* L. amnigena* DSM 4486^T^	4,370,208	52.9	4,070	PDDA01000000
* L. jeotgali* PFL01^T^	4,603,334	54.2	4,243	CP018628
*Pluralibacter*				
* P. gergoviae* NBRC 105706^T^	5,662,775	58.6	5,176	BCZS00000000
*Pseudescherichia*				
* P. vulneris* NBRC 102420^T^	4,374,581	56.4	4,196	BBMZ00000000
*Raoultella*				
* R. ornithinolytica* NBRC 105727^T^	5,533,930	55.7	5,099	BCYR00000000
* R. planticola* ATCC 33531^T^	5,668,028	55.8	5,237	JMPP00000000
*Xenorhabdus*				
*X. nematophila* ATCC 19061^T^	4,587,917	44.3	3,754	FN667742

Note

Data are from the GenBank/EMBL/DDBJ databases.
